# Innovating the way medical emergency teams function, can this lead to better patient outcomes? an attempt to understand this better

**DOI:** 10.1186/2197-425X-3-S1-A144

**Published:** 2015-10-01

**Authors:** R Deb, MI Alam, AT Patil, S Razvi, KS Reddy, R Paul

**Affiliations:** Apollo Hospitals, Critical Care, Hyderabad, India; Apollo Hospitals, Pediatric Pulmonology, Hyderabad, India; Apollo Hospitals, Internal Medicine, Hyderabad, India

## Introduction

The increasing demand for hospital beds, an ageing population and shorter hospital stays have resulted in increased patient acuity and greater risk of clinical deterioration. This can have life threatening consequences which include in-hospital cardiac arrest, unplanned admission to the intensive care units (ICU) and death.

**Rapid Response Systems (RRS) or Medical emergency Teams (MET)** are hospital wide systems that have been developed to provide a safety net for ward patients who suddenly deteriorate and develop complex care needs that may be outside the scope of clinical ward staff knowledge and skills.

## Objectives

To try and evaluate if a **pro-active MET approach, started from October 2013,** based on daily hospital rounds of select patient groups, to try and identify at-risk patients, is more effective, when compared with the traditional, physiological-criteria based alert system, in reducing the overall Code Blue numbers in a tertiary care hospital in India.

## Methods

In an effort to try and reduce the overall numbers of Code blues in our hospital, a JCI certified 550 bed Hospital in India, daily rounds of the hospital, by a team comprising of a MET physician and two Nurse supervisors were started and during the rounds, which started after 5 pm in the evening, we tried to visit a certain group of patients who, we felt, were the most vulnerable to deterioration, which could result in a life threatening situation. These patients were,

1.All patients on oxygen therapy, respiratory support therapy in the wards (Bi-pap / C-pap)

2.All ICU transfer outs during the day

3.All post operative cases

4.All in-patients undergoing dialysis

5.All patients receiving chemotherapy

6.All patients who had received procedural sedation during the day

7.Patients in the BMT ward

8.Patients in the Tracheostomy ward, and

9.Any other patient the staff had a “bad feeling” about.

These patients would then either be shifted to the ICU for further management, or interventions like labs, dialysis, escalation in therapy would be initiated and followed up stringently.We have attempted to compare the code blue rates for a period of one year before this system was put in place with the numbers since then and tried to analyze if this pro-active approach to a traditional concept can help reduce Codes in a setting like ours.

## Results

An analysis of this data has shown that a pro-active approach has helped us in reducing the number of Codes at our Hospital over the period of a year, when compared to the earlier reactive approach.

**(Group A- October 2012 to september 2013 vs Group B- October 2013 to September 2014).**

## Conclusions

The results of our comparison have encouraged us to try to come up with a proforma wherein we streamline this process with the belief that this will not only help us in better identifying patients with the potential to deteriorate, thereby decreasing adverse events, but also in better analyzing our data.

**Figure 1:**
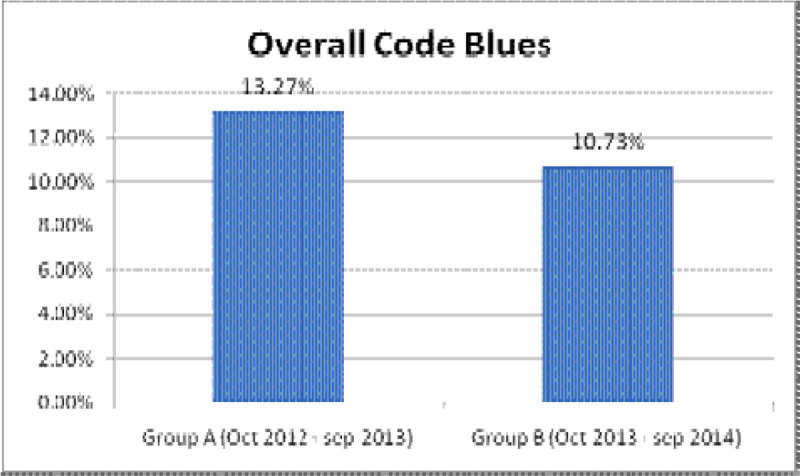
**Overall code blues in Group A vs Group B.**

**Figure 2:**
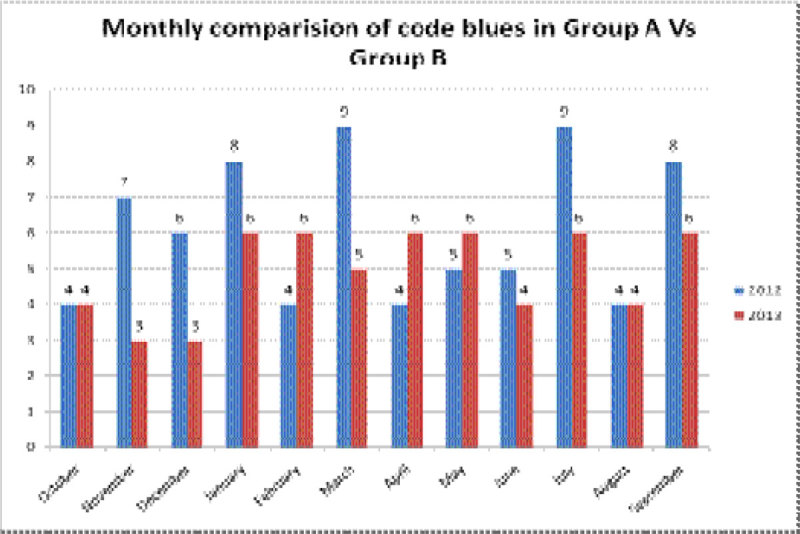
**Monthwise Comparison of Code Blues.**

